# Single‐Cell Transcriptomics Reveals Biomarkers for NK Cell Dysfunction in Endometriosis‐Associated Immune Dysregulation

**DOI:** 10.1155/mi/9028037

**Published:** 2026-02-03

**Authors:** Wangshu Li, Kexin Zhu, Bowen Xu, Juan Nie, Fang Wang, Aziz ur Rehman Aziz, Xiaohui Yu, Daqing Wang, Chunfang Ha

**Affiliations:** ^1^ Department of Key Laboratory of Pediatric and Female Malignant Tumors, Dalian Women and Children’s Medical Group, Dalian, China; ^2^ Department of Gynecology, Ningxia Autonomous Region People’s Hospital, Yinchuan, China; ^3^ Department of Gynecology, General Hospital of Ningxia Medical University, Yinchuan, China, nxmu.edu.cn

**Keywords:** biomarkers, endometriosis, immune dysregulation, natural killer cells, single-cell RNA sequencing

## Abstract

**Background:**

Endometriosis (EM) is associated with immune dysregulation, while dysfunction of natural killer (NK) cells is regarded as a key mechanism underlying immune escape and the persistent growth of ectopic lesions.

**Method:**

This study used single‐cell RNA sequencing (scRNA‐seq) on lesions from three patients with EM and on three normal endometrium samples and integrated these data with three bulk RNA‐seq datasets from GEO (GSE105765, GSE7305, and GSE6364). Seurat, Monocle, limma, least absolute shrinkage and selection operator (LASSO), and support vector machine recursive feature elimination (SVM‐RFE) were used for cell clustering, trajectory inference, differential expression analysis, and feature selection. Immune‐cell composition and pathway activity were evaluated with CIBERSORT and GSVA. Gene expression was validated by qPCR, and cell migration and invasiveness were assessed using wound healing and Transwell assays.

**Result:**

scRNA‐seq resolved 11 clusters assigned to eight major cell types. By integrating pseudotime features with bulk data, 20 differentially expressed genes (DEGs) were prioritized, and machine‐learning analyses identified three key genes: granulysin (GNLY), perforin 1 (PRF1), and ENTPD1. The three‐gene model showed good discrimination in the training set and two external validation cohorts (AUCs 0.84, 0.67, and 0.77, respectively). GNLY and PRF1 were predominantly expressed in NK cells and CD8^+^ T cells and correlated with activation signatures, whereas ENTPD1 was highly expressed in endometrial stromal cells and enhanced their migratory and invasive capacities. ENTPD1 may contribute to disease via adenosine signaling–mediated modulation of NK–cell function. In silico analyses also nominated candidate agents targeting this pathway, including resveratrol, ibuprofen, and danazol.

**Conclusion:**

This study highlights the central role of NK–cell dysfunction in EM pathogenesis and proposes GNLY, PRF1, and ENTPD1 as potential molecular diagnostic biomarkers. Notably, ENTPD1 appears to have dual functions, including immunomodulation and promotion of stromal cell migration, which promotes lesion formation. These findings provide a mechanistic rationale and actionable targets for earlier screening and targeted therapy in EM.

## 1. Introduction

Endometriosis (EM) is a chronic inflammatory disorder characterized by the ectopic growth of endometrial‐like tissue outside the uterine cavity, affecting ~10% of women of reproductive age worldwide [1, 2]. The disease often causes severe pelvic pain and infertility, markedly impairing quality of life and placing a substantial burden on healthcare systems. Diagnosis is frequently delayed, with an average lag of 7–12 years after symptom onset [3, 4]. Despite extensive investigation, the pathogenesis of EM remains unclear. Growing evidence indicates that immune dysregulation plays a pivotal role in disease initiation and progression [5].

Among the immune abnormalities documented in EM, dysfunction of natural killer (NK) cells has emerged as a major pathophysiological mechanism [6, 7], whereby NK cells—principal effectors of the innate immune system—conduct immune surveillance and eliminate virus‐infected, transformed, and mislocalized or metastatic cells, which maintain tissue homeostasis [8]. Under physiological conditions, these cells recognize and clear endometrial cells that enter the peritoneal cavity via retrograde menstruation, which prevents ectopic implantation and subsequent proliferation [9]. In EM, however, NK–cell impairment constitutes what amounts to a composite phenotype in which cytotoxic activity is diminished, receptor‐expression profiles are reprogrammed, and chemotactic responses are attenuated [10–12], such that peritoneal NK cells from affected women display marked reductions in both killing and migration, with the chemotactic defect persisting throughout the menstrual cycle [13]. The result is a permissive immune milieu in which ectopic endometrial cells that would otherwise be eliminated achieve immune escape, which allows them to survive, implant, and expand at extrauterine sites [14].

Current evidence points to complex cellular and molecular interactions within the local microenvironment as the basis for NK–cell dysfunction. Estrogen‐mediated mechanisms may attenuate NK activity by downregulating CD16 and granzyme B expression; moreover, crosstalk between endometrial stromal cells and other immune cells can further compromise NK–cell function [15]. Nevertheless, the precise molecular pathways and key genes governing NK–cell dysregulation have yet to be fully delineated.

This study applies an integrated multiomics framework to investigate the molecular mechanisms underlying NK–cell dysfunction and its contribution to EM progression. By combining single‐cell RNA sequencing (scRNA‐seq) with bulk transcriptomic datasets, we construct a cellular atlas of endometriotic lesions and identify molecular signatures associated with immune dysregulation. Through in vitro functional assays, we further delineate the role of ENTPD1 in promoting endometrial stromal cell migration and invasion, thereby providing mechanistic insight into EM pathogenesis and proposing potential therapeutic targets to enable earlier intervention and personalized treatment strategies.

## 2. Methods

### 2.1. Data Acquisition and Preprocessing

In this study, a self‐constructed scRNA‐seq dataset comprising three EM lesions and three homologous endometrial samples was obtained from ethically approved patient tissues for which informed consent was obtained. Samples were enzymatically lysed, single‐cell suspensions prepared and assessed for cell viability (>85%), followed by library construction using the 10x Genomics Chromium Single Cell 3′ v3 kit; sequencing was performed on the Illumina NovaSeq 6000 platform at an average of ~50,000 reads/cell. Raw data were aligned (GRCh38/hg38), barcode demultiplexed, UMI de‐duplicated, and expression matrix generated using CellRanger (v6.1.1).

Bulk RNA‐seq data were downloaded from GEO database, including GSE105765 (eight normal endometrium and nine EM), GSE7305 (10 normal and 10 EM), and GSE6364 (16 normal and 21 EM), which were used as the training, validation, and test sets, respectively. The data were normalized and preprocessed using the “affy” R package (v1.70.0) and the RMA algorithm.

### 2.2. Single‐Cell Data Analysis

Quality control criteria included: (1) 200–5000 genes detected per cell; (2) genes detected in < 3 cells were excluded, (3) mitochondrial genes accounted for < 30% of the total genes, and (4) UMIs > 100. Subsequent analyses were performed using the Seurat R package (v4.0.5): logarithmic normalization of the data. The data were log‐normalized, and 2000 highly variable features were screened by “FindVariableFeatures”; the data were normalized by “Find Integration Anchors” and “IntegrateData” to correct for batch effects; after standardization, principal component analysis (PCA) was performed, and the first 40 principal components were used in “FindNeighbors,” “FindClusters” (resolution 0.2), and “FindVariableFeatures” (resolution 0.2). The first 40 principal components were used for “FindNeighbors” and “FindClusters” (resolution 0.2) clustering and visualized by “RunUMAP.”

Cell types were annotated based on CellMarker 2.0 database marker genes. Cluster‐specific differentially expressed genes (DEGs) were identified by “FindAllMarkers” with thresholds: log_2_FC > 0.5, min.pct = 0.5, corrected *p*  < 0.05. Subpopulations of immune cells were extracted for centralized analysis. Functional enrichment was performed using clusterProfiler (v4.0.5) with org.Hs.eg.db (v3.13.0) for Gene Ontology (GO) and Kyoto Encyclopedia of Genes and Genomes (KEGG) analysis.

Proposed temporal trajectories were inferred using Monocle3 (v1.0.0): Seurat objects were converted to CellDataSet, “learn_graph” was used to infer the structure of the trajectory, and ‘order_cells’ was applied to order cells in pseudotime. The ‘graph_test’ was then used to detect dynamic genes (*q*‐value < 0.05).

### 2.3. Bulk RNA Analysis and Biomarker Screening

DEGs were screened in the bulk dataset using limma (v3.48.3) (log_2_FC > 1, corrected *p*  < 0.05) and intersected with scRNA‐seq differential genes. Feature selection was performed using support vector machine recursive feature elimination (SVM‐RFE; e1071 v1.7–9) with the least absolute shrinkage and selection operator (LASSO; glmnet v4.1–2), combined with 10‐fold cross‐validation. Hub genes were validated by univariate and multivariate logistic regression. Column line plots were constructed based on rms (v6.2–0), pROC (v1.18.0) plotted receiver operating characteristic (ROC) curves, and decision curve analysis (DCA) (v2.0) carried out DCA.

Immune infiltration was estimated using CIBERSORT (v1.03); the relationship between genes and immune cells was assessed by Spearman correlation analysis. The Hallmark and KEGG pathways were scored using GSVA (v1.40.1), and GSEA was implemented using clusterProfiler, with the criteria of standardized enrichment score (NES) > 1 and corrected *p*  < 0.05. Protein Interaction Networks (PPIs) were constructed based on STRING (v11.5) and were used for DCA in Cytoscape (v3.9.1) for visualization. Potential drugs were screened by the Comparative Toxicogenomic Database (CTD).

### 2.4. Cell Culture and Transfection

Human uterine stromal cells (HUSCs) were purchased from ShangEn Biotechnology (Wuhan, China; Cat. No. SNP‐H371). Immortalized human endometrial stromal cells (ihESCs, mimicking EM–like behavior [16]) were purchased from YiMo Biotechnology (Xiamen, China; cat. no. IM‐H540). Both cell lines were authenticated by the suppliers using short tandem repeat profiling and supplied with certificates of analysis confirming identity and mycoplasma‐free status. Cells were used within 10 passages of receipt. Cells were cultured in complete endometrial stromal cell medium (provided by the respective suppliers) supplemented with 10% fetal bovine serum and 100 U/mL penicillin with 100 μg/mL streptomycin and incubated at 37°C, 5% CO_2_.

OE experiments were performed using Lipofectamine 3000 (Invitrogen) transfected with pcDNA3.1‐ENTPD1 or empty vector (OE‐NC); knockdown experiments were performed with siRNA targeting ENTPD1 (si‐ENTPD1) or negative control (si‐NC) (final concentration, 50 nM; sequence: si‐ENTPD1). Transfection efficiency was routinely >80% for ihESCs and 70%–85% for HUSCs. Transfection efficiency was verified by qPCR 48 h after transfection.

### 2.5. Quantitative Real‐Time PCR (qRT‐PCR)

Total RNA was extracted using TRIzol (Invitrogen) and reverse transcribed using the PrimeScript RT kit (Takara); quantification was performed on a QuantStudio 5 system (Applied Biosystems) using SYBR Green Master Mix. The primers were as follows: ENTPD1: Forward 5′‐AGGTGTGGATATCAGCCTGTA‐3′, Reverse 5′‐CTTCTCTCCGAGATCCCTTCC‐3′;GNLY: Forward 5′‐ATATCGTCTCCCAGATGCACT‐3′, Reverse 5′‐AGCTTTCCTCTCCAAGTTGAT‐3′;PRF1: Forward 5′‐CCACTCACAGGCAGCCAA‐3′, Reverse 5′‐GGAGATGAGCCTGTGGTAAG‐3′;GAPDH: Forward 5′‐GAAGGTGAAGGTCGGGAGTC‐3′, Reverse 5′‐GAAGATGGTGATGGGATTTC‐3′.

### 2.6. Transwell Invasion Assay

The transfected ihESCs were inoculated at 5 × 10^4^ cells in the upper chamber of a Matrigel‐coated Transwell (pore size 8 μm, Corning) with serum‐free medium; the lower chamber was added with medium containing 20% FBS. After 24 h of incubation, the infiltrating cells were fixed with 4% paraformaldehyde and stained with 0.1% crystal violet; five random fields of view were taken and counted under the microscope (200×). The experiment was completed in three independent replicates.

### 2.7. Scratch Healing Experiment

Transfected ihESCs were cultured in 6‐well plates to 90% fusion, and “wounds” were formed by making scratches with a sterile lance tip and continued to be cultured in serum‐free medium; imaging was performed using an inverted microscope at 0 h and 24 h. The results were obtained by using ImageJ (v.1) and the inverted microscope at 0 h and 24 h, respectively. The width of the scratch was measured using ImageJ (v1.53), and the wound healing rate was calculated: healing rate (%) = (initial width ‐ 24 h width)/initial width × 100. The experiment was repeated three times independently.

### 2.8. Statistical Analysis

Statistical analyses were performed using R v4.3.2 and GraphPad Prism 9.0. Differential expression analysis was conducted using the Wilcoxon rank‐sum test with Benjamini–Hochberg false discovery rate (FDR) correction; adjusted *p*  < 0.05 and |log_2_FC| > 0.5 were considered significant. For in vitro experiments involving multiple groups, one‐way ANOVA followed by Tukey’s post hoc test was used; for two‐group comparisons, unpaired two‐tailed Student’s *t*‐test or Mann–Whitney *U* test was applied. Multiple comparisons in functional assays were corrected using Bonferroni adjustment. All reported *p*‐values are adjusted where multiple testing was performed, and adjusted *p*  < 0.05 was considered statistically significant.

## 3. Results

### 3.1. Construction and Characterization of Single‐Cell Transcriptomic Landscape in EM

To delineate the cellular landscape of EM, we performed scRNA‐seq on ectopic lesions from three patients and paired eutopic endometrium from three control individuals. After stringent quality control filtering, cells exhibiting a negative correlation between mitochondrial gene content and total transcript counts, alongside a positive correlation between detected gene number and UMI counts, were retained (Figure 1A). Among the 2000 highly variable genes identified, prominent expression of cytotoxic and proinflammatory mediators (GNLY, IL1B, CXCL8, S100A9, and LYZ) was observed (Figure 1B).

Figure 1Single‐cell quality control and clustering. (A) Violin plots of quality metrics: nFeature_RNA, nCount_RNA, and percent.mt before and after filtering. (B) Scatterplot of variable features vs. nonvariable. (C) Elbow plot for PCA dimensions. (D) JackStraw plot for significant PCs. (E) Stacked bar plot showing the proportion of cells from each sample origin across clusters. (F) Bar plot illustrating the number of cells per cluster. (G) UMAP visualization of cells colored by cluster identification. (H) Hierarchical clustering heatmap of average expression of cluster‐specific marker genes across major cell types. (I) UMAP visualization of all cells annotated with major cell types. (J) Dot plot showing the expression of selected marker genes across major cell types.

**Figure:**
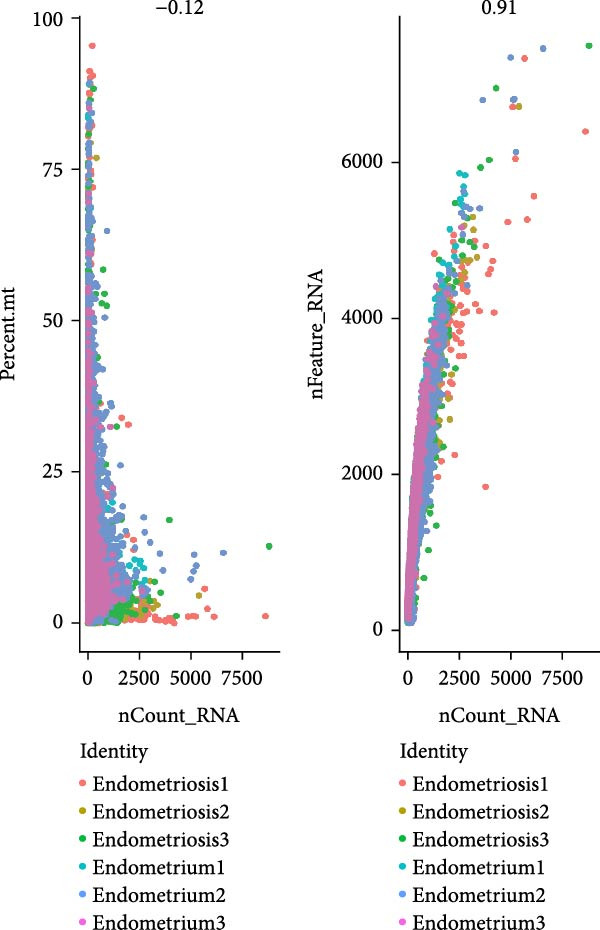
(A)

**Figure:**
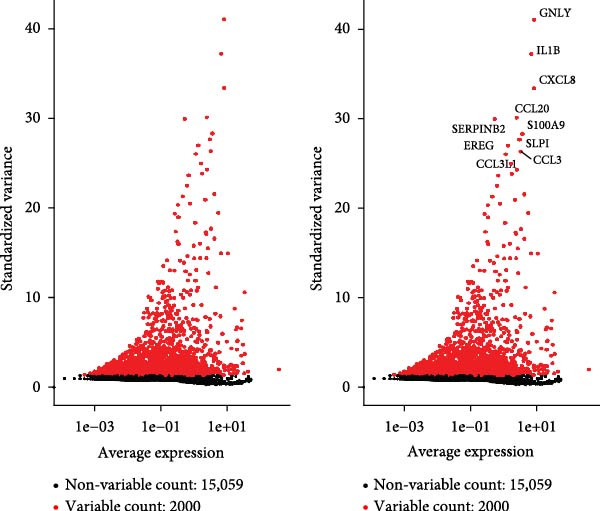
(B)

**Figure:**
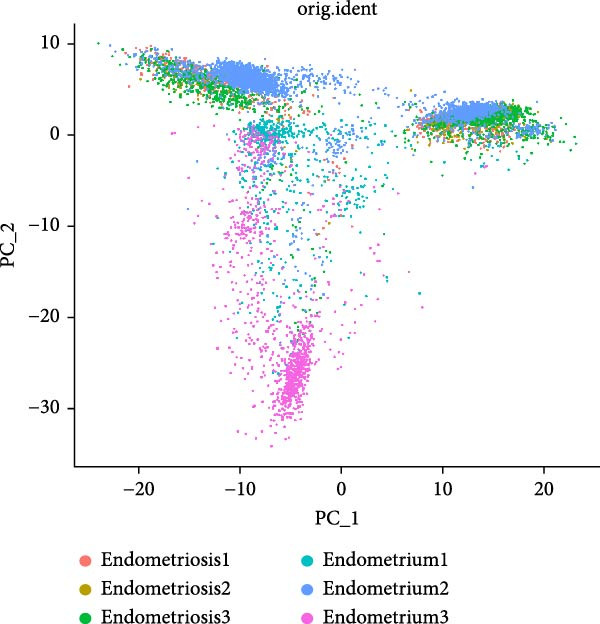
(C)

**Figure:**
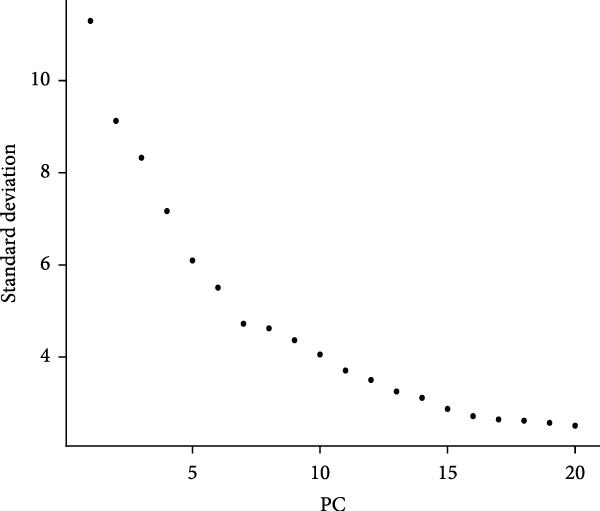
(D)

**Figure:**
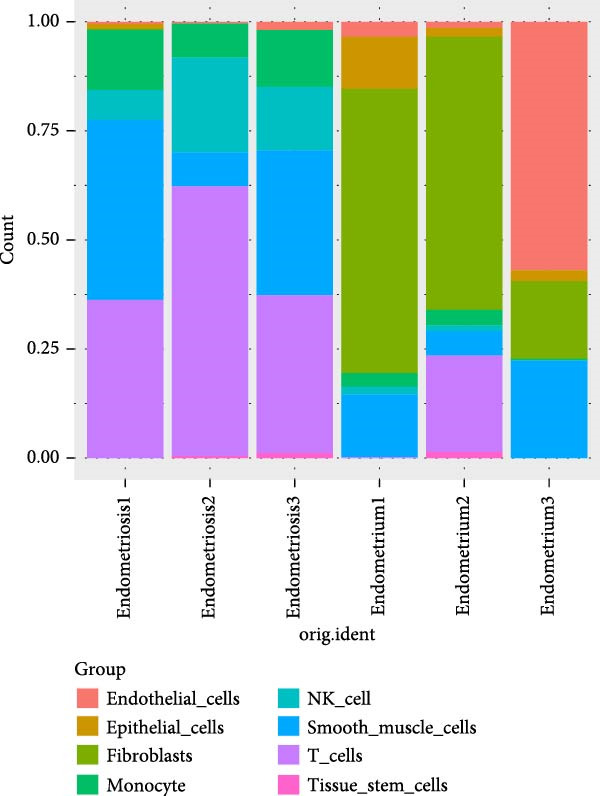
(E)

**Figure:**
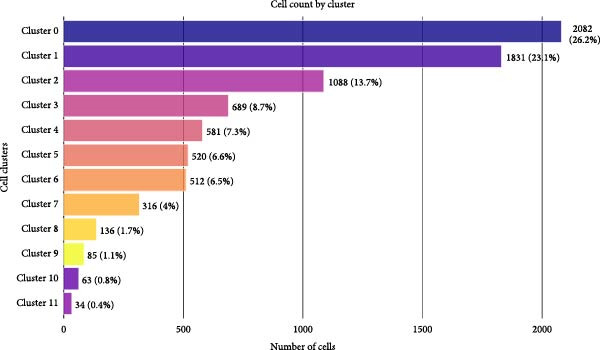
(F)

**Figure:**
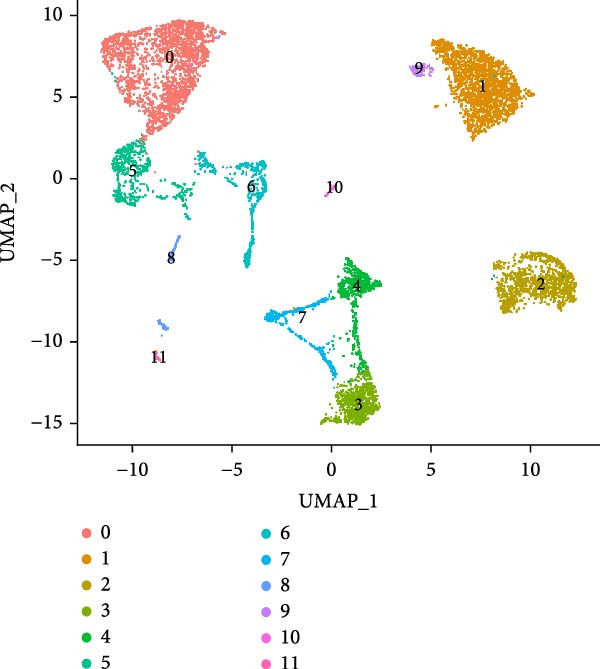
(G)

**Figure:**
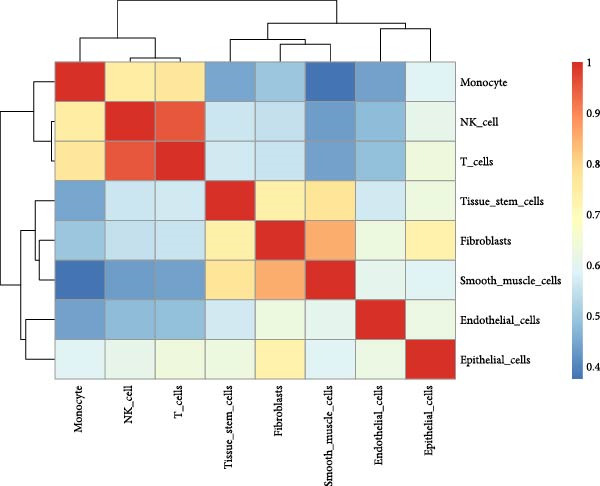
(H)

**Figure:**
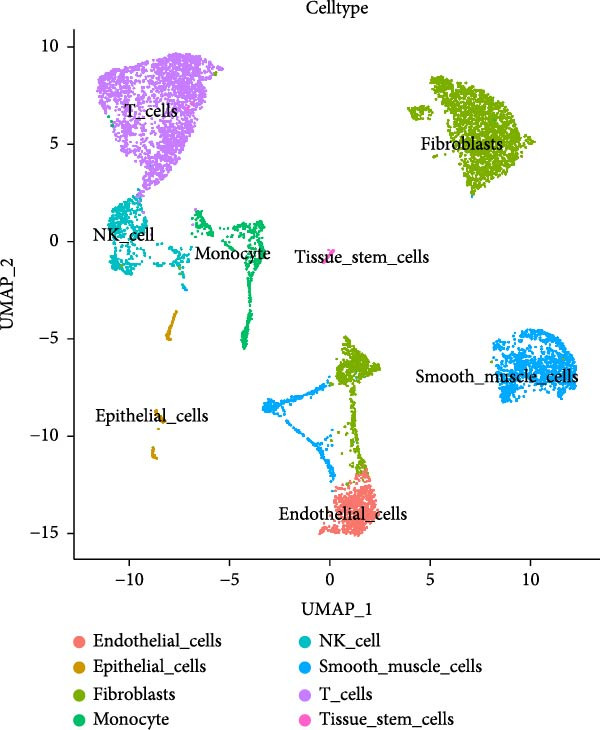
(I)

**Figure:**
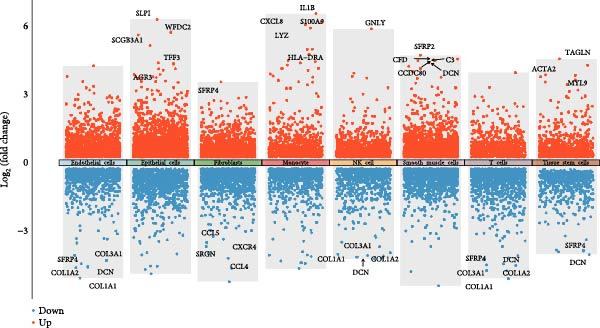
(J)

PCA revealed clear separation between endometriotic and control samples (Figure 1C), with the elbow plot guiding selection of the top 20 principal components for downstream analysis (Figure 1D). Unsupervised clustering at resolution 0.8 identified 11 transcriptionally distinct clusters (clusters 0–11) that were visualized on UMAP (Figure 1G) and annotated into eight major cell types based on established canonical markers and SingleR reference mapping (Figure 1H,I): fibroblast cells (clusters 0, 1, and 3), epithelial cells (cluster 2), endothelial cells (cluster 4), T cells (cluster 5), NK cells (cluster 6), macrophages (cluster 7), smooth muscle cells (cluster 8), and tissue stem cells (cluster 9), with minor clusters 10–11 representing low‐quality or transitional populations.

Marked differences in cellular composition were evident between EM and control samples (Figure 1E,F). Differential expression analysis across cell types highlighted upregulation of inflammatory and cytotoxic molecules (SLPI, WFDC2, IL1B, CXCL8, S100A9, LYZ, and GNLY) predominantly in immune subsets, whereas extracellular matrix and Wnt pathway modulators (COL1A1, COL1A2, DCN, SFRP2, and SFRP4) were downregulated in fibroblast and smooth muscle compartments (Figure 1J). These findings collectively establish a comprehensive single‐cell atlas revealing profound immune activation and stromal remodeling in endometriotic lesions.

### 3.2. Molecular Signatures and Functional Enrichment of Identified Cell Populations

The accuracy of cell type annotations was validated through spatial distribution of signature genes. Epithelial cells specifically expressed EPCAM, while endothelial cells were enriched for vascular markers. Smooth muscle cells were characterized by TAGLN and ACTA2 expression; fibroblasts by high levels of COL1A1, COL1A2, and DCN; and NK/T cells by cytotoxic markers NKG7, GNLY, GZMB, and PRF1. Monocytes showed distinctive LYZ expression (Figure 2A–C). GO and pathway analyses revealed significant enrichment in immune response, granulocyte chemotaxis, cytokine–receptor interaction, leukocyte differentiation, and ECM organization (Figure 2D,E).

Figure 2Cell subtypemarkers and functional enrichment. (A) Feature plots of selected markers on UMAP. (B) Dot plot of percent expressed and average expression for top markers. (C) Heatmap of *Z*‐scores for markers across cell types, clustered by identity. (D) GO enrichment dot plot showing terms like leukocyte migration (gene ratio and p.adjust). (E) KEGG enrichment dot plot highlighting pathways such as cytokine–receptor interaction.

**Figure:**
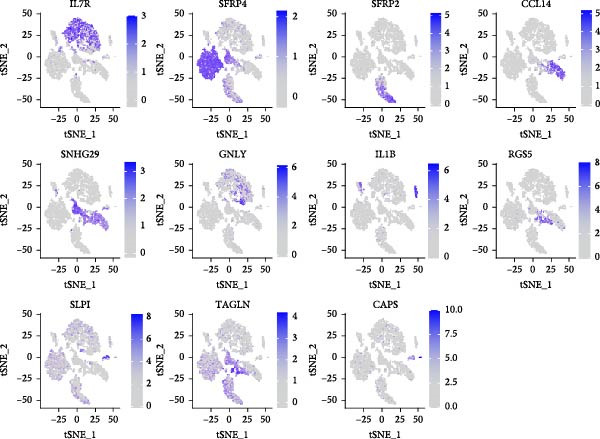
(A)

**Figure:**
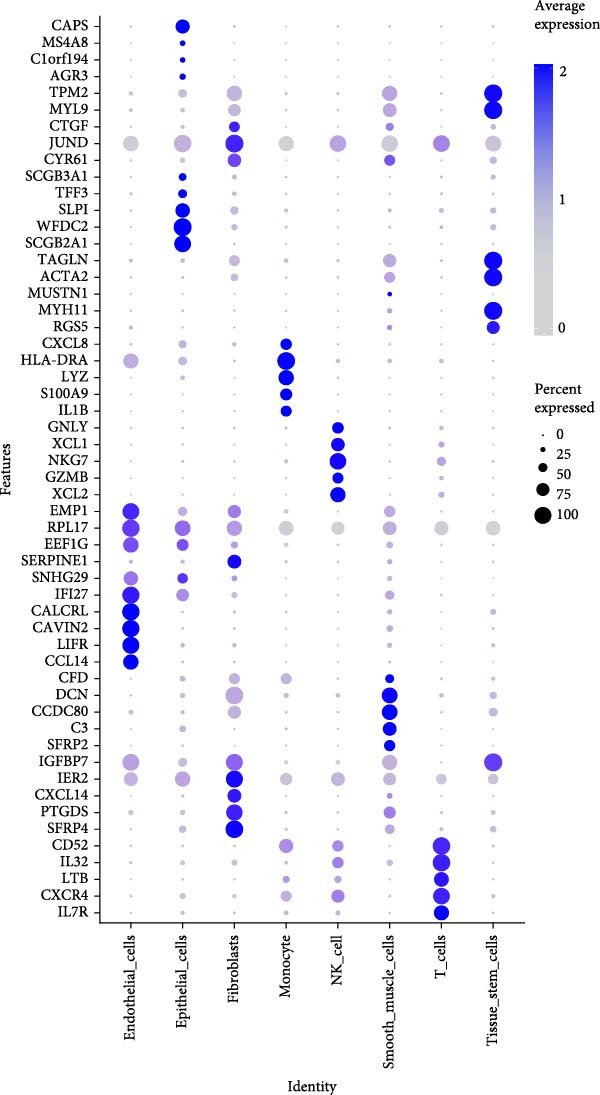
(B)

**Figure:**
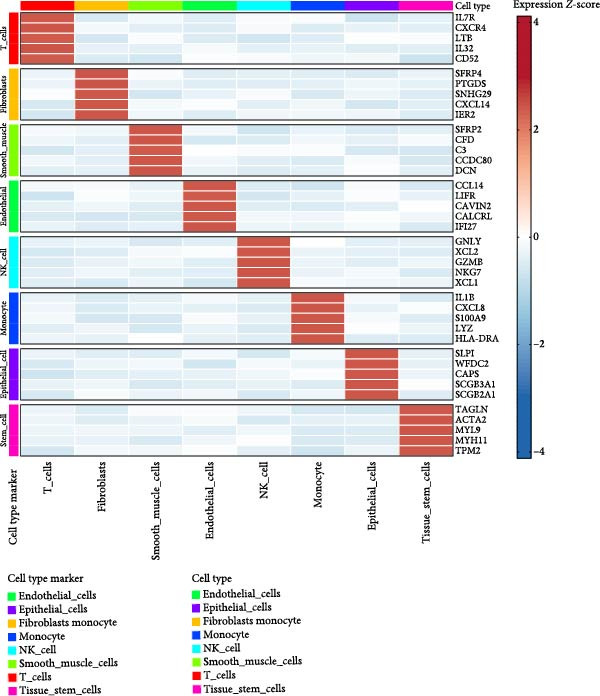
(C)

**Figure:**
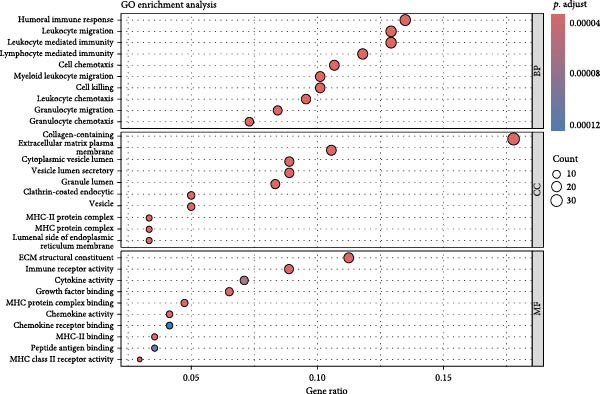
(D)

**Figure:**
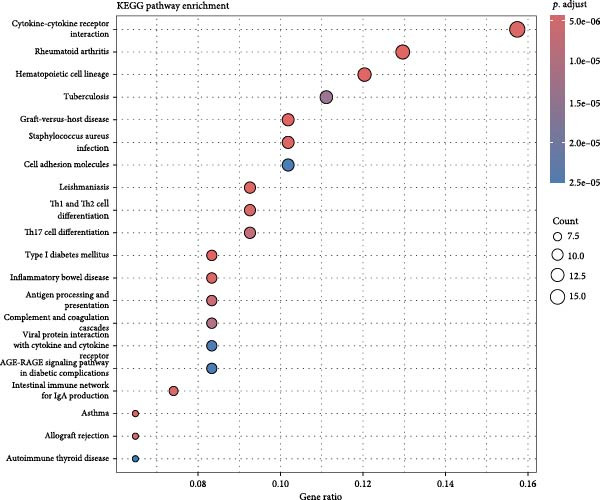
(E)

### 3.3. Trajectory Inference Reveals Disease‐Associated Cellular Dynamics

Pseudotemporal ordering of cells uncovered continuous state transitions in the disease microenvironment (Figure 3A,B,D). Along the inferred trajectory, immune effector molecules progressively increased, while ECM/fibrosis–related genes (COL1A1, COL1A2, DCN, and SPARCL1) gradually decreased, indicating an “immune activation‐matrix remodeling” continuum during lesion formation (Figure 3C). Integration of single‐cell differential genes with trajectory‐associated markers identified 361 key genes (Figure 3E), whose cell type‐specific expression patterns are depicted in Figure 3F.

Figure 3Pseudotime trajectoryanalysis. (A, B) UMAP trajectories split by group (control vs. EM). (C) Pseudotime‐colored UMAP with branches. (D) Cell type distribution along trajectory. (E) Venn diagram of scDEGs and dynamic trajectory markers. (F) Heatmap of top differentially expressed genes along pseudotime.

**Figure:**
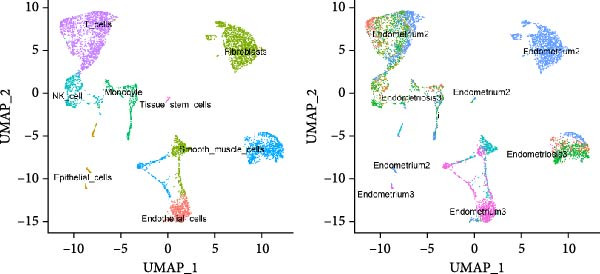
(A)

**Figure:**
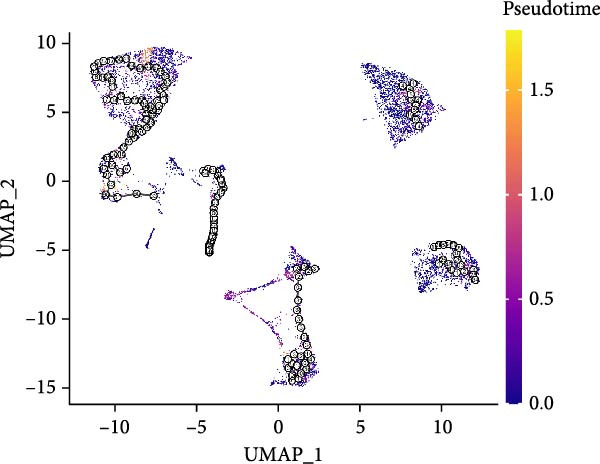
(B)

**Figure:**
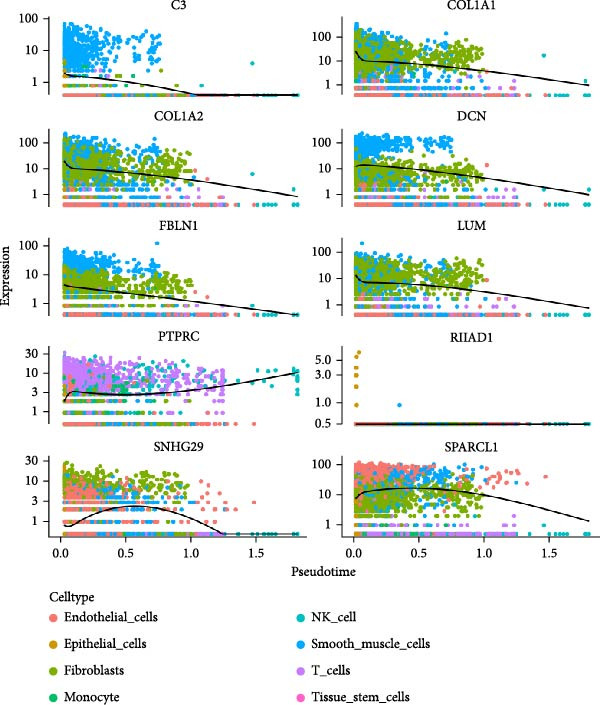
(C)

**Figure:**
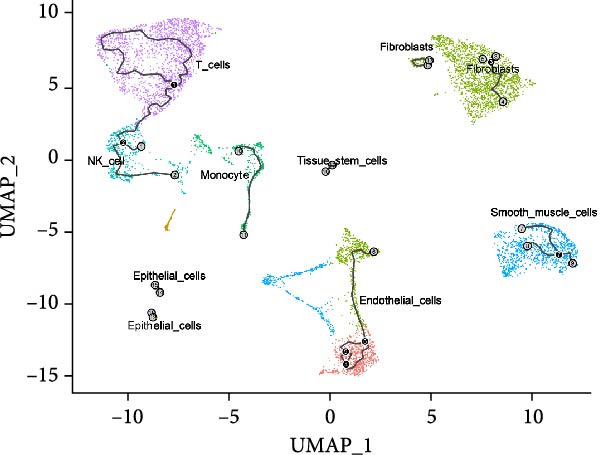
(D)

**Figure:**
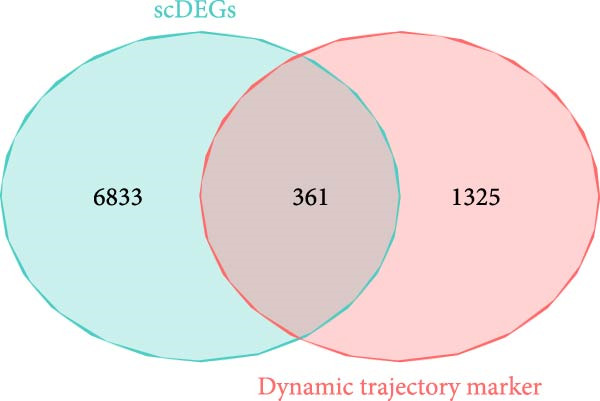
(E)

**Figure:**
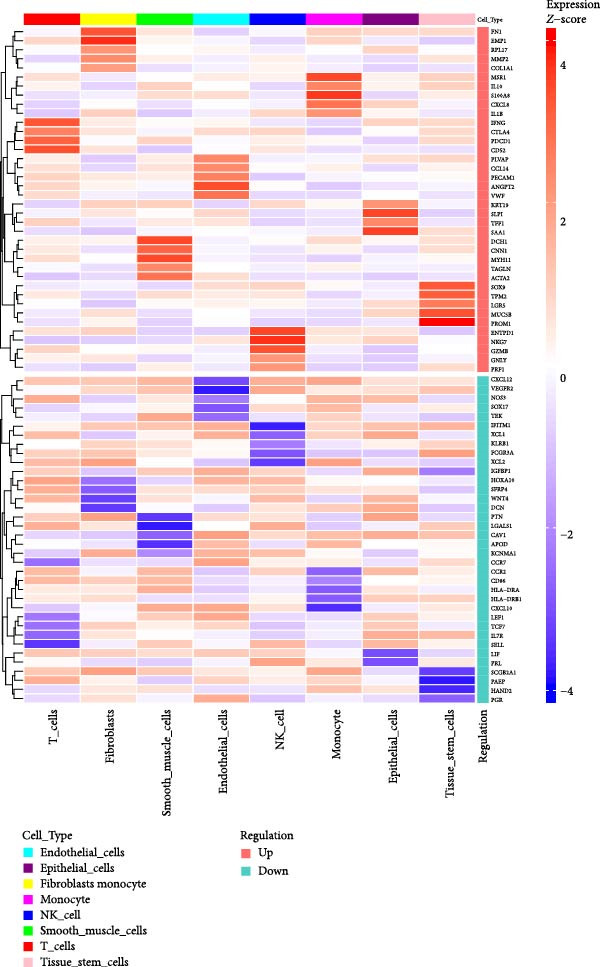
(F)

### 3.4. Cross‐Platform Validation Identifies Robust Disease Markers

To validate our single‐cell findings, we analyzed two independent bulk RNA‐seq cohorts (GSE105765 and GSE7305), identifying numerous DEGs (Figure 4A,B). Intersection with single‐cell‐derived key genes yielded 20 consistently dysregulated candidates (Figure 4D), showing reproducible expression patterns across datasets (Figure 4C,E). Protein‐protein interaction network analysis positioned these genes as central hubs in immune regulation and cytokine signaling (Figure 4F).

Figure 4Bulk transcriptome differential expression. (A, B) Volcano plots for GSE105765 (281 DEGs) and GSE7305 (1260 DEGs), with up/down/no‐change points. (C) Heatmap of top DEGs clustered by sample group (normal vs. tumor). (D) Venn diagram of key genes overlapping across datasets. (E) Heatmap of 20 differentially expressed genes. (F) PPI network with functional modules.

**Figure:**
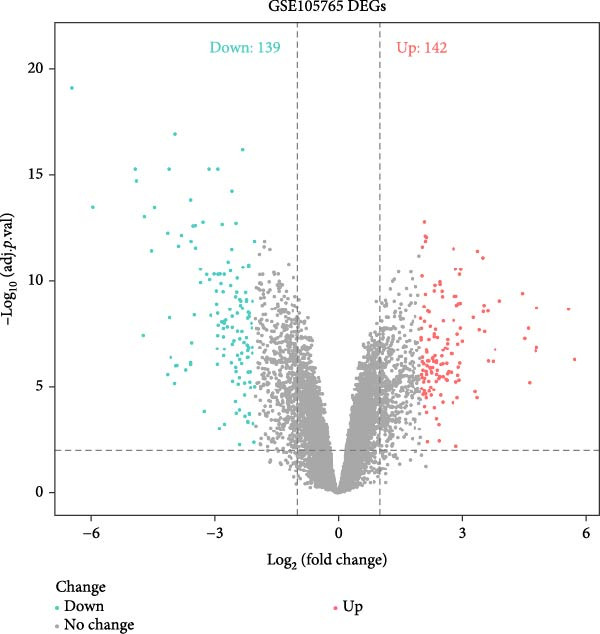
(A)

**Figure:**
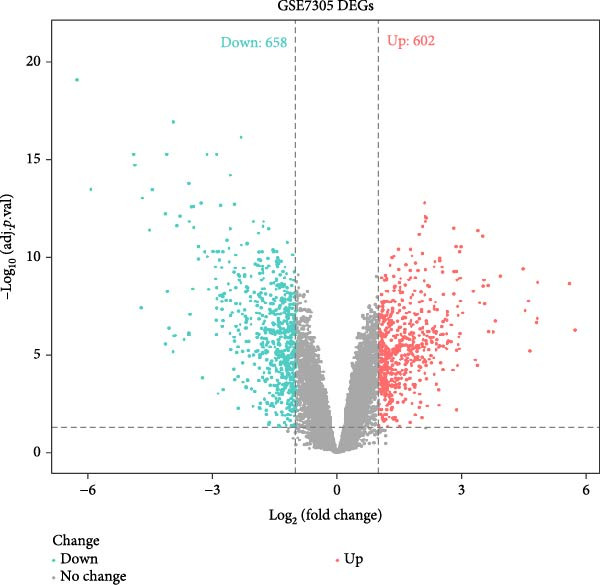
(B)

**Figure:**
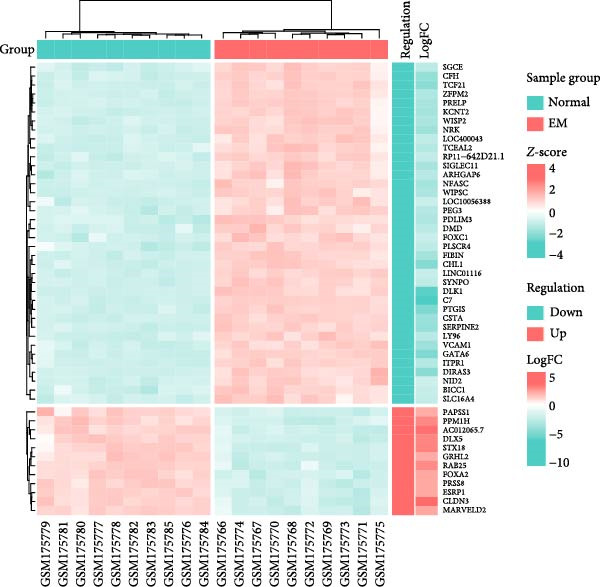
(C)

**Figure:**
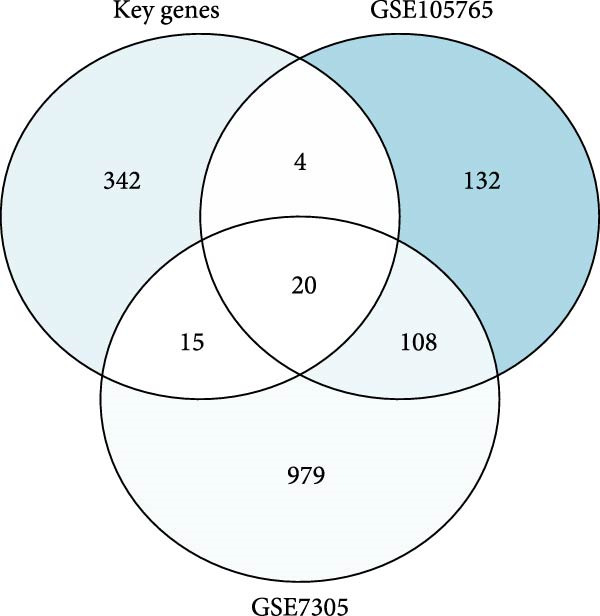
(D)

**Figure:**
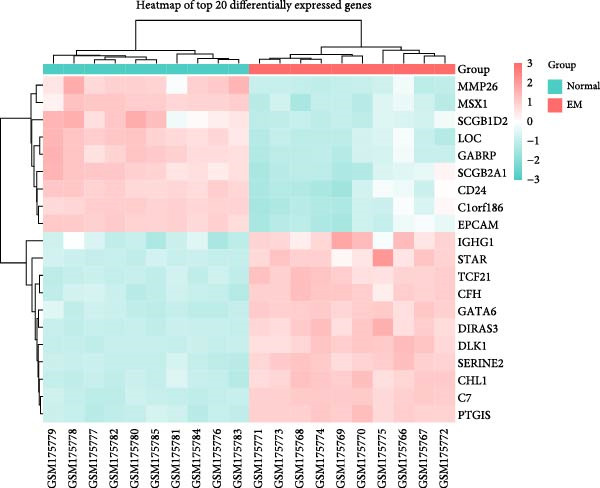
(E)

**Figure:**
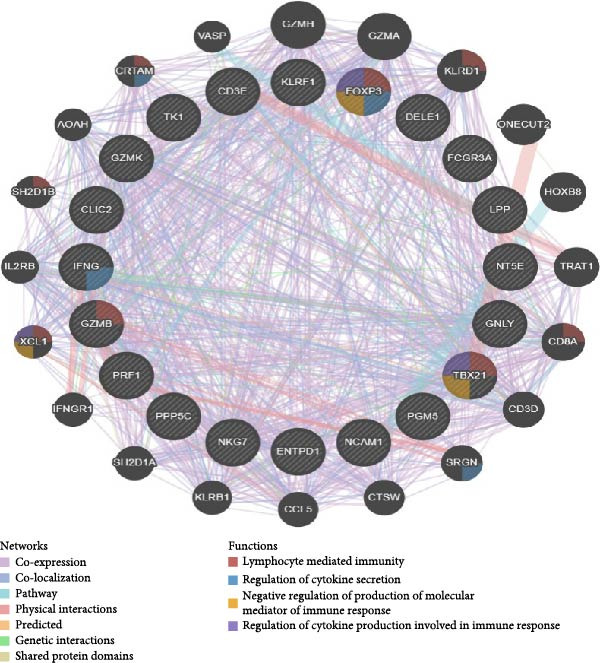
(F)

### 3.5. Cytotoxicity‐Related Genes Demonstrate Strong Discriminative Power

Univariate analysis identified NKG7, GZMB, SCGB2A1, EPCAM, ENTPD1, GNLY, PRF1, PTGIS, GATA6, and STAR as significantly associated with disease status (Figure 5A). These genes exhibited marked expression differences between endometriotic and control tissues (Figure 5B), with single‐gene ROC curves demonstrating excellent discriminative ability (AUC ≥ 0.80 for most genes; Figure 5C).

Figure 5Gene expressionvalidation and risk assessment. (A) Hazard ratio table with forest plot for candidate genes. (B) Boxplots of gene expression in eutopic vs. ectopic tissues. (C) ROC curves for individual genes.

**Figure:**
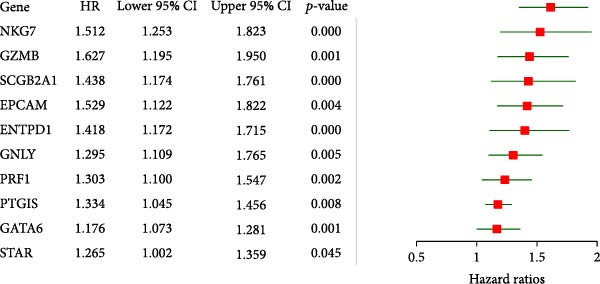
(A)

**Figure:**
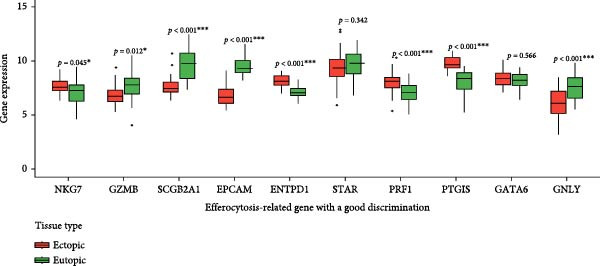
(B)

**Figure:**
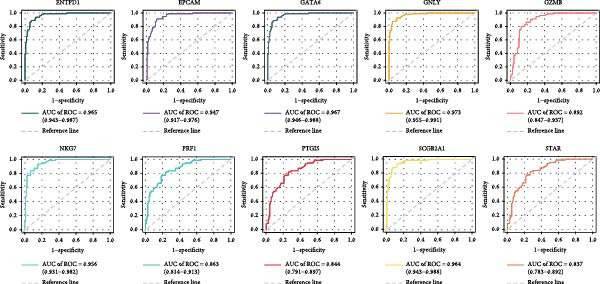
(C)

### 3.6. Machine Learning‐Based Feature Selection Establishes a Three‐Gene Diagnostic Signature

SVM‐RFE identified an optimal 8‐gene subset achieving ~90% cross‐validation accuracy (Figure 6A). LASSO regression further refined the feature set, revealing stable coefficient paths (Figure 6B,C) and identifying genes with the highest weights (Figure 6D). Based on these analyses, we developed a nomogram incorporating GNLY, PRF1, and ENTPD1 for EM risk assessment (Figure 7A).

Figure 6Machine learning‐based biomarker selection. (A) SVM‐RFE feature selection curve showing cross‐validation accuracy peaking at 5 features (0.9898). (B) LASSO cross‐validation curve with 10‐fold CV, minimizing binomial deviance at *λ* = 0.02734. (C) LASSO coefficient paths for candidate genes, with vertical lines indicating selected *λ*. (D) Normalized expression bar plots of the final three hub genes (ENTPD1, GNLY, and PRF1) across samples.

**Figure:**
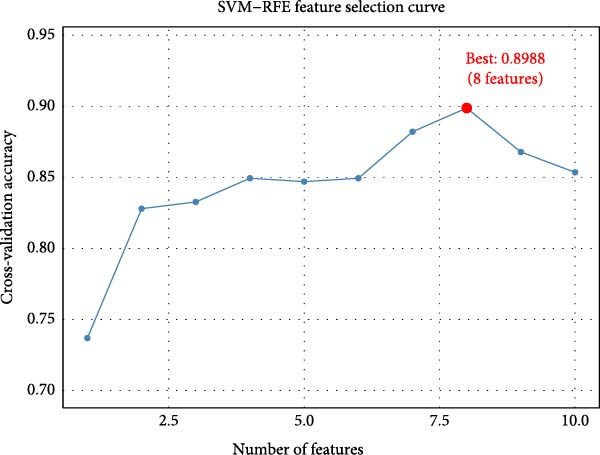
(A)

**Figure:**
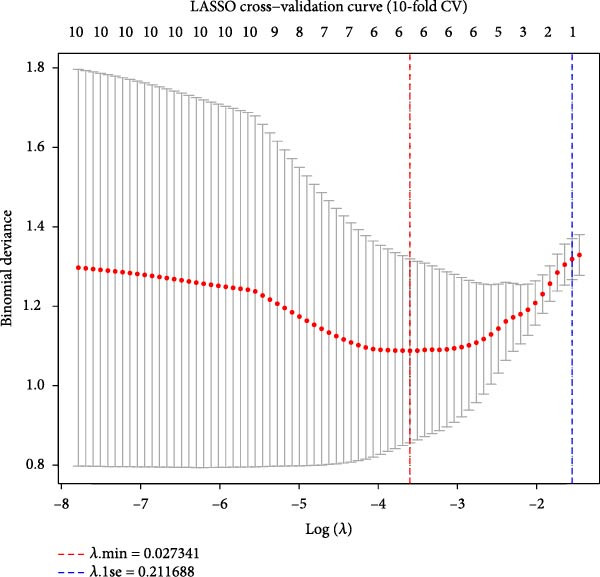
(B)

**Figure:**
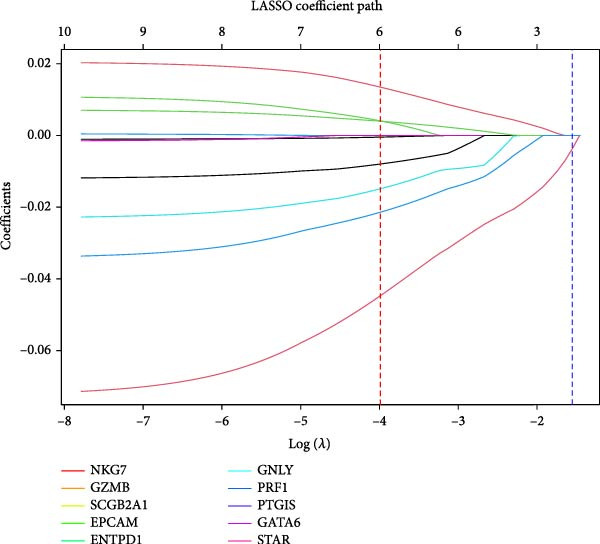
(C)

**Figure:**
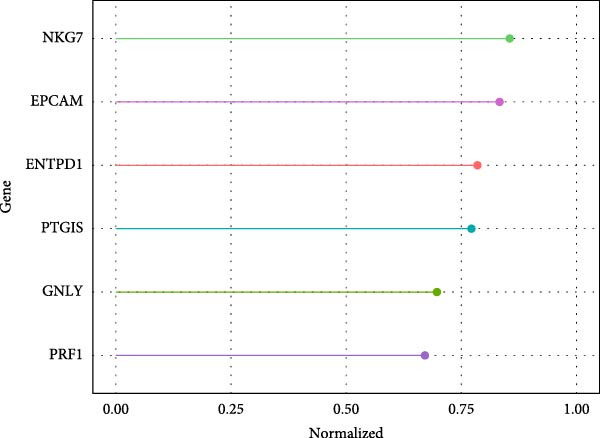
(D)

Figure 7Diagnostic nomogram and performance evaluation. (A) Nomogram for EM risk prediction based on three‐gene signature, with points scale. (B) Calibration curve comparing predicted vs. observed risks (with apparent and bias‐corrected lines). (C) DCA curves for train, test, and validation cohorts showing net benefit. (D) ROC curves for external cohorts.

**Figure:**
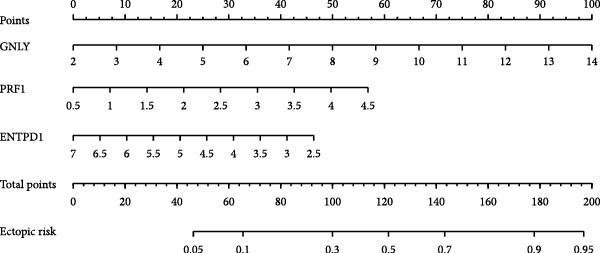
(A)

**Figure:**
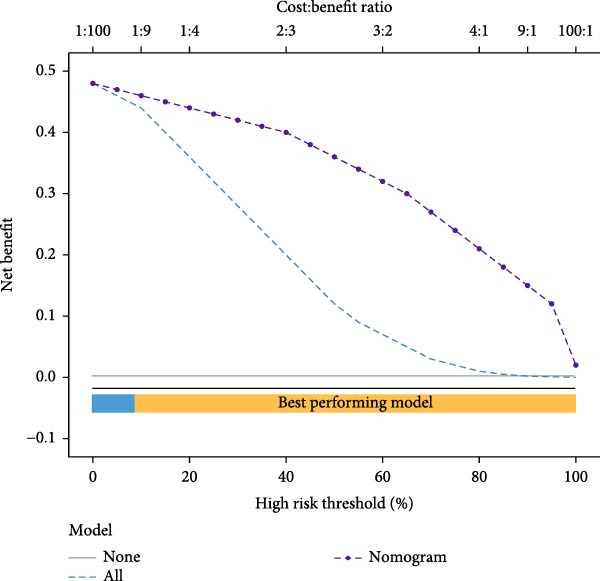
(B)

**Figure:**
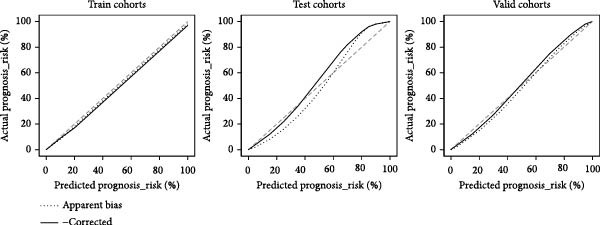
(C)

**Figure:**
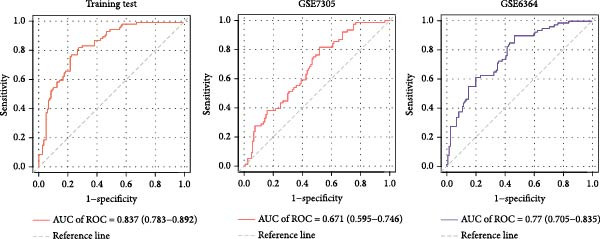
(D)

### 3.7. Diagnostic Nomogram Constructed and Validated for EM

To provide a quantitative tool for EM risk prediction, we developed a nomogram incorporating the three hub genes (GNLY, PRF1, and ENTPD1) using the training cohort (GSE105765) (Figure 7A). In the nomogram, lower expression of GNLY and PRF1 (reflecting impaired cytotoxic activity) and higher ENTPD1 expression contributed to higher total points and greater predicted probability of EM.

DCA demonstrated that the nomogram conferred superior clinical net benefit across a wide range of threshold probabilities (10%–90%) compared with treat‐all or treat‐none strategies, confirming its clinical utility (Figure 7B).

Calibration curves revealed excellent agreement between nomogram‐predicted probabilities and actual EM occurrence in the training cohort (GSE105765), internal validation cohort (GSE7305), and independent external test cohort (GSE6364), with bias‐corrected lines closely aligning with the ideal reference line (Figure 7C; Table S1).

Receiver operating characteristic (ROC) analysis further confirmed robust discriminatory performance, yielding AUC values of 0.837 (95% CI 0.783–0.892) in the training cohort, 0.671 (95% CI 0.595–0.746) in the validation cohort (GSE7305), and 0.777 (95% CI 0.705–0.835) in the test cohort (GSE6364) (Figure 7D). These results collectively validate the three‐gene nomogram as a reliable and clinically applicable diagnostic tool for EM.

### 3.8. Immune Infiltration Landscape Correlates With Signature Genes

Applied CIBERSORT deconvolution to the integrated bulk RNA‐seq cohorts. This analysis revealed profound remodeling of the immune microenvironment, with significant differences in the proportions of multiple immune cell subpopulations between EM lesions and normal endometrium (Figure 8A, C). Notably, EM samples exhibited a marked reduction in resting NK cells (*p*  < 0.001) and activated CD^8+^ T cells, alongside increased fractions of M2 macrophages (*p*  < 0.01), resting mast cells, and neutrophils (Figure 8A, C), consistent with an immunosuppressive and profibrotic milieu that favors lesion persistence. GSVA of immune‐related pathway signatures across major cell types identified from scRNA‐seq further highlighted suppressed cytotoxic activity in EM (Figure 8B). Cytotoxic effector programs, including “activated NK cells,” “CD8^+^ T cell memory/activation,” and “gamma‐delta T cells,” displayed markedly lower pathway scores in EM–associated clusters compared to controls, whereas signatures associated with alternative macrophage polarization and regulatory T cell activity were relatively enriched (Figure 8B).

Figure 8Immune infiltration patterns and gene‐immune correlations in endometriosis. (A) Stacked bar plots showing proportions of 22 immune cell types estimated by CIBERSORT in control and EM samples. (B) Correlation matrix of immune cell types, illustrating coupled relationships. (C) Boxplots of selected immune cell proportions. (D, E) Spearman correlation analyses: lollipop plots and scatterplots demonstrating associations between hub genes (GNLY, PRF1, and ENTPD1) and immune cells.

**Figure:**
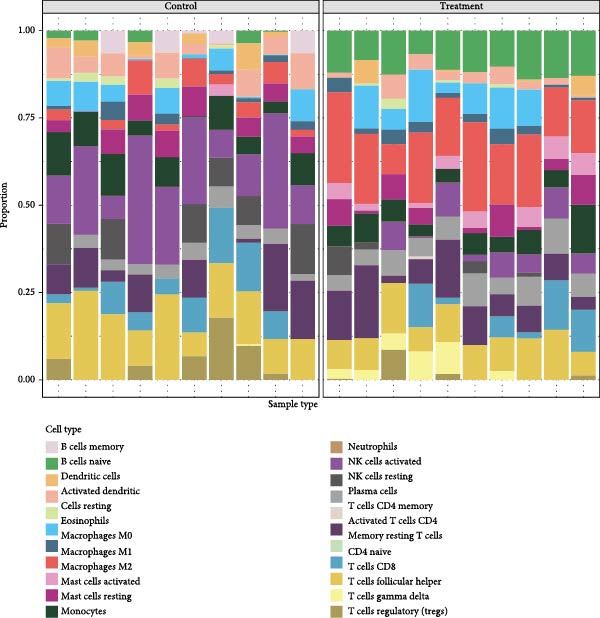
(A)

**Figure:**
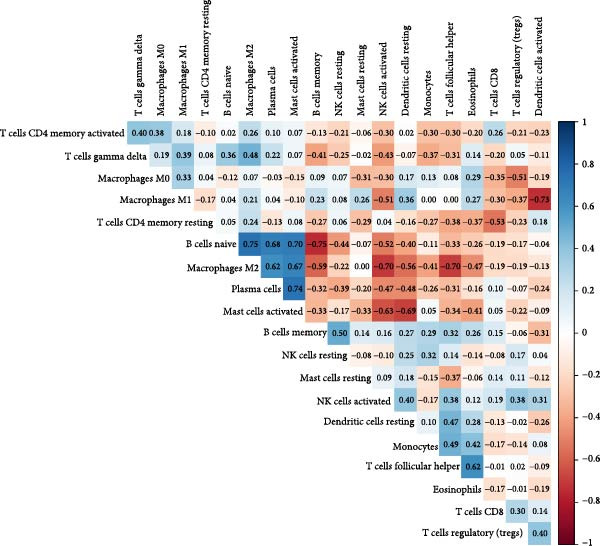
(B)

**Figure:**
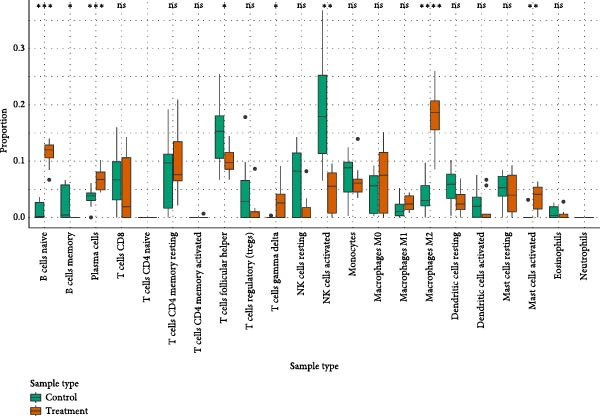
(C)

**Figure:**
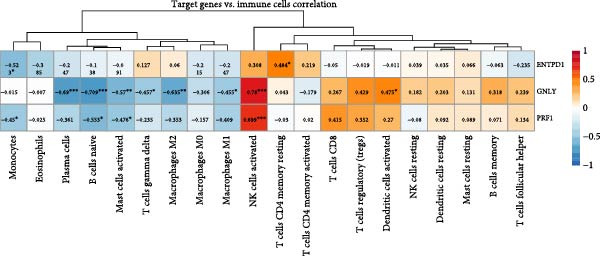
(D)

**Figure:**
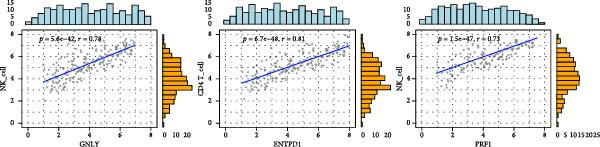
(E)

Spearman correlation analysis revealed distinct immune infiltration patterns for the three hub genes (Figure 8D,E). GNLY and PRF1 showed strong positive correlations with activated NK cells (*r* ≈ 0.70–0.78) and activated/memory CD^8+^ T cells (*r* = 0.45–0.65) but negative correlations with M2 macrophages and regulatory T cells. In bulk samples, both genes strongly correlated with NK–cell infiltration (GNLY: *r* = 0.78, *p* = 5.8 × 10^−42^; PRF1: *r* = 0.73, *p* = 1.5 × 10^−47^). In contrast, ENTPD1 exhibited the strongest positive correlation with resting NK cells (*r* = 0.781, *p*  < 0.001), monocytes, eosinophils, and M2 macrophages while displaying weaker/negative correlations with activated cytotoxic lymphocytes and the highest correlation with CD^8+^ T cell infiltration (*r* = 0.81, *p* = 6.7 × 10^−48^). Thus, GNLY and PRF1 primarily mark impaired cytotoxic activity in EM, whereas ENTPD1, despite predominant stromal expression, associates with CD^8+^ T cell infiltration and suppressive myeloid populations, which may contribute to NK–cell dysfunction via adenosine‐mediated immunomodulation.

### 3.9. Single‐Cell Resolution Mapping Analysis of Immune Cell‐Specific Expression

To validate the immune cell‐specific expression patterns of the hub genes identified from bulk RNA‐seq analysis, we performed independent scRNA‐seq analysis using a publicly available dataset of EM and related tissues (GSE213216), which includes endometriomas, ectopic and eutopic endometrium, unaffected ovary, and EM–free peritoneum. As shown in (Figure 9A,C), UMAP dimensionality reduction revealed 18 distinct cell clusters, which were successfully annotated into major cell populations within the disease microenvironment, including epithelial cells, fibroblasts, endothelial cells, macrophages, monocytes, CD^4^+ T cells, CD^8^+ T cells, B cells, and NK cells. The cellular composition analysis indicated that fibroblasts were the most abundant cell type (26%), followed by B cells (15%), monocytes (13%), NK cells (13%), endothelial cells (12%), CD^8^+ T cells (10%), macrophages (8%), and CD^4^+ T cells (3%), highlighting the relatively high infiltration and potential functional importance of NK cells in the EM–associated microenvironment.

Figure 9Independent scRNA‐seq validation of hub genes. (A–C) UMAPs from GSE213216: clustering, cell type annotation, and proportion pie chart. (D–F) Violin plots of hub gene expression across cell types. (G) Dot plot quantifying average expression and percent expressed for ENTPD1, GNLY, and PRF1 in immune subsets.

**Figure:**
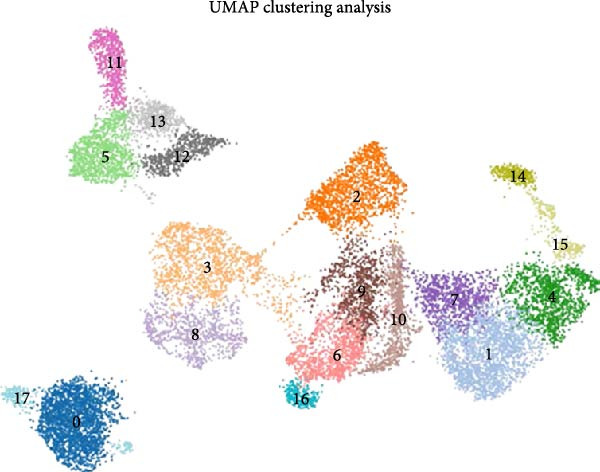
(A)

**Figure:**
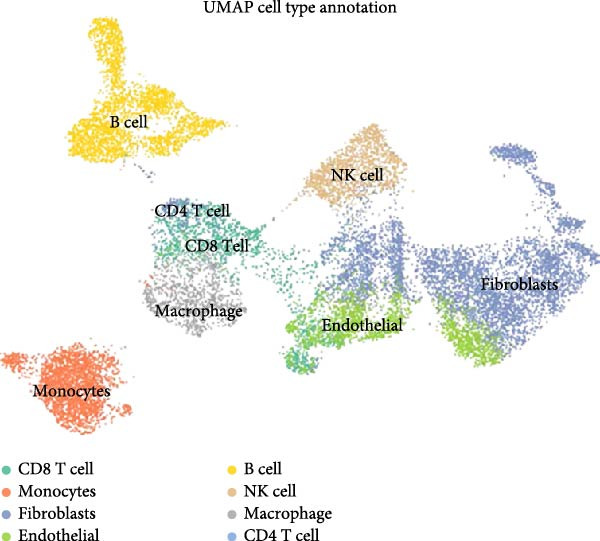
(B)

**Figure:**
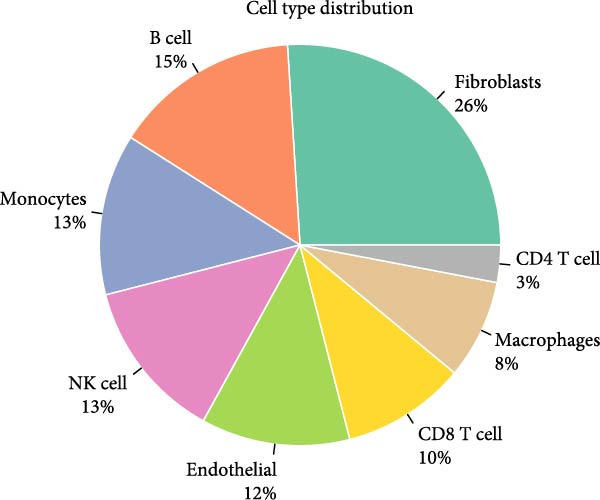
(C)

**Figure:**
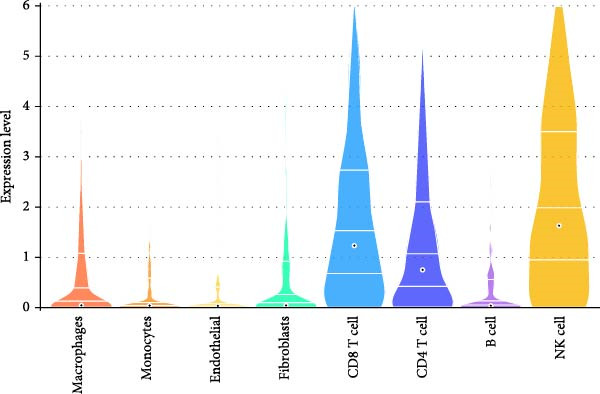
(D)

**Figure:**
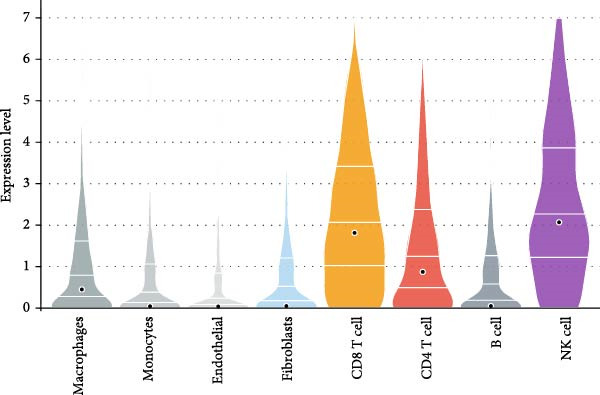
(E)

**Figure:**
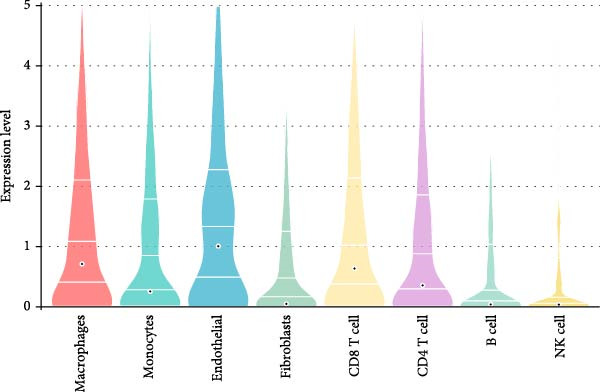
(F)

**Figure:**
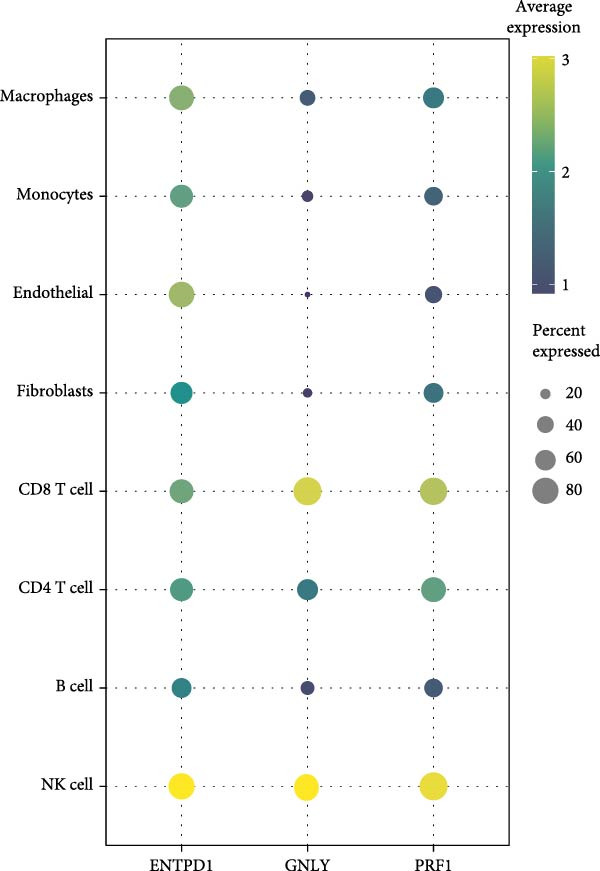
(G)

Violin plots demonstrated clear cell‐type‐specific expression of the hub genes (Figure 9D,F). ENTPD1 (CD39) was predominantly expressed in myeloid subsets (macrophages and monocytes) and CD^8^+ T cells, with minimal expression in NK cells and B cells. In contrast, GNLY and PRF1 exhibited the highest expression levels in NK cells and CD^8^+ T cells, consistent with their established roles in cytotoxic function. The dot plot further quantified the average expression and percentage of cells expressing these genes across immune subsets (Figure 9G). Notably, NK cells displayed the highest average expression of GNLY and PRF1, as well as the broadest distribution of expressing cells among cytotoxic effectors, despite their moderate abundance. ENTPD1 showed strong expression primarily in macrophages, monocytes, and endothelial cells, supporting its role in immunosuppressive adenosine signaling. These single‐cell resolution findings independently confirm the immune cell‐specific expression patterns of the hub genes, underscoring the dominant cytotoxic potential of the NK cell population and the immunosuppressive contributions from myeloid and endothelial compartments in the EM microenvironment.

### 3.10. Pathway Enrichment and Drug Repurposing Opportunities

Hallmark and KEGG enrichment analyses, combined with GSEA, revealed that genes coexpressed with our signature were significantly enriched in interferon response, IL2‐STAT5 signaling, TNF*α*‐NF*κ*B signaling, inflammatory response, hypoxia, complement cascade, and Th1/Th2 differentiation, while oxidative phosphorylation was suppressed (Figure 10A–C). Drug–gene interaction network analysis identified multiple clinically available compounds potentially targeting ENTPD1/GNLY/PRF1, including resveratrol, ibuprofen, indomethacin, rofecoxib, danazol, mifepristone, and progesterone (Figure 10D), suggesting therapeutic repurposing opportunities.

Figure 10Functional enrichment and drug prediction. (A) GSVA dot plot of top Hallmark terms (e.g., mesenchymal transition and enrichment score). (B) GSVA heatmap clustered by gene expression levels. (C) GSEA bar plot of KEGG pathways. (D) Protein–drug interaction network with nodes for hub genes and drugs.

**Figure:**
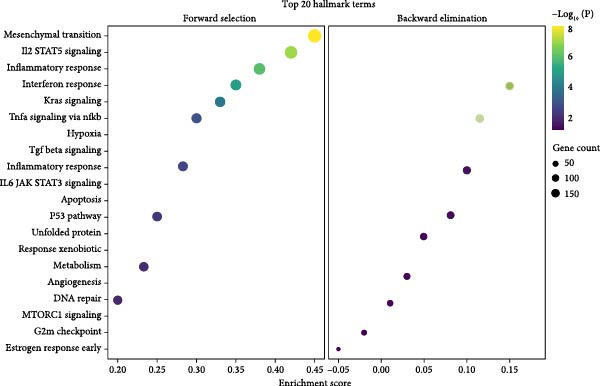
(A)

**Figure:**
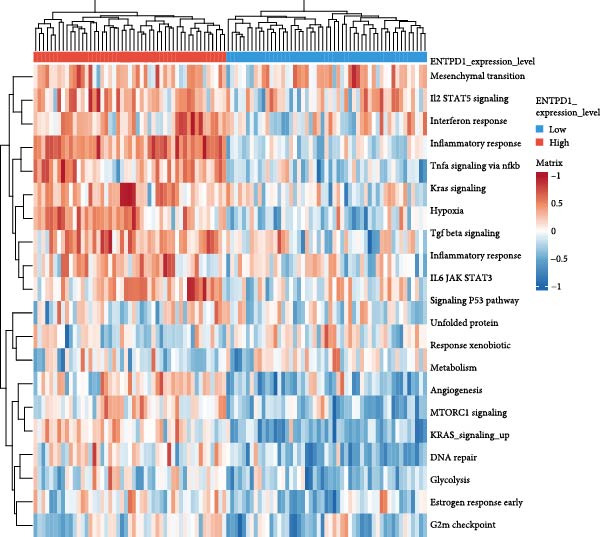
(B)

**Figure:**
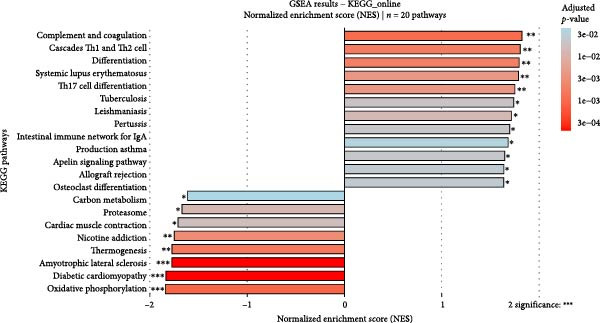
(C)

**Figure:**
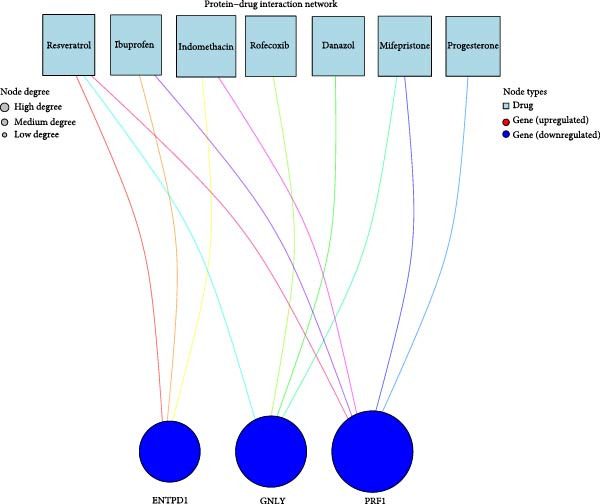
(D)

### 3.11. ENTPD1 Promotes Endometrial Stromal Cell Migration

To functionally validate our computational findings, we first compared transcript levels of the three signature genes in primary HUSCs and ihESCs. While GNLY and PRF1 showed no significant differences between cell types, ENTPD1 was significantly elevated in ihESCs (2.0‐fold,  ^∗∗^ *p*  < 0.001; Figure 11A), consistent with our bulk and single‐cell analyses.

Figure 11In vitro functional validation of ENTPD1. (A) qRT‐PCR bar plots showing relative mRNA levels of ENTPD1, GNLY, and PRF1 in HUSCs vs. ihESCs ( ^∗∗∗^ *p*  < 0.001 for ENTPD1). (B) Wound healing assay images (0/24 h) for OE‐NC, OE‐ENTPD1, si‐NC, and si‐ENTPD1 groups, with right bar plot quantifying closure rate ( ^∗∗^ *p*  < 0.01 and  ^∗∗∗^ *p*  < 0.001). (C) Transwell invasion assay images (crystal violet staining) for the same groups, with right bar plot of invaded cell counts ( ^∗∗^ *p*  < 0.01 and  ^∗∗∗^ *p*  < 0.001).

**Figure:**
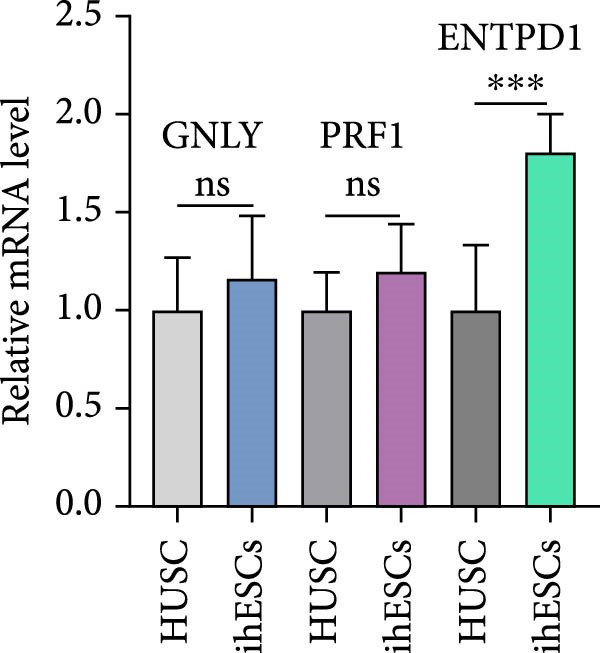
(A)

**Figure:**
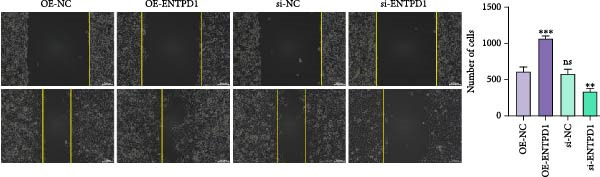
(B)

**Figure:**
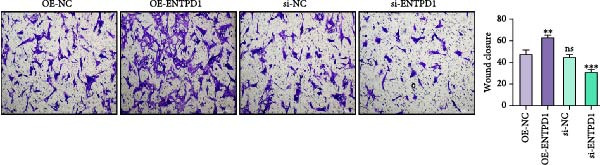
(C)

Cell function experiments demonstrated that ENTPD1 OE (OE‐ENTPD1) significantly enhanced wound healing and cell migration, whereas ENTPD1 knockdown (si‐ENTPD1) produced opposite effects (Figure 11B,C). Specifically, OE‐ENTPD1 increased scratch closure by 30%–40% compared to control (OE‐NC, *p*  < 0.01), with a 1.7–fold increase in migrating cells (*p*  < 0.001; Figure 11B). Conversely, si‐ENTPD1 reduced scratch closure by 30%–40% relative to si‐NC ( ^∗∗^ *p*  < 0.001) and decreased migrating cell numbers (*p*  < 0.01; Figure 11B). Transwell migration/invasion assays corroborated these findings: ENTPD1 overexpression significantly increased transmembrane cell numbers, while silencing markedly inhibited migration (Figure 11C; OE‐ENTPD1 *p*  < 0.01 and si‐ENTPD1  ^∗∗^ *p*  < 0.001).

Collectively, ENTPD1 demonstrates clear promigratory and wound healing functions in endometrial stromal cells, while GNLY and PRF1 show no significant changes in stromal cells, supporting their cell type‐specific expression in cytotoxic immune cells. These experimental validations, combined with our multicohort, multiscale analyses, establish ENTPD1 as a key functional molecule in EM pathogenesis and a promising therapeutic target.

## 4. Discussion

EM is a multifactorial gynecological disorder characterized by ectopic endometrial‐like tissue accompanied by chronic pelvic pain and infertility [17]. Beyond retrograde menstruation, convergent evidence identifies immune dysfunction—particularly attenuated NK cell cytotoxicity—as a driver of lesion persistence and progression [7]. Multiple studies report reduced peripheral and peritoneal NK cytotoxicity in affected individuals, facilitating immune evasion and sustained proliferation of ectopic endometrial cells within the peritoneal cavity [18, 19]. Mechanistically, impaired function involves imbalanced expression of activating and inhibitory NK receptors together with an immunosuppressive cytokine milieu [20, 21].

This study applied a multiomics framework to delineate NK dysfunction and its interface with stromal biology in EM. scRNA‐seq generated a high‐resolution cellular atlas of endometriotic lesions that, integrated with bulk transcriptomes, yielded a three‐gene diagnostic signature (GNLY, PRF1, and ENTPD1). Functional perturbation established an effector role for ENTPD1 in endometrial stromal cell migration and invasion, situating immune–stromal crosstalk as a central process in disease progression. These results outline immune escape and local microenvironmental remodeling as interlinked axes with translational relevance for early intervention and individualized therapy.

### 4.1. Molecular Basis of Cytotoxic Immune Dysfunction: GNLY and PRF1

GNLY and PRF1 are core cytotoxic effectors of NK cells and cytotoxic T lymphocytes that mediate target‐cell death through pore formation and granzyme delivery (for example, GZMB) [22, 23]. At single‐cell resolution, GNLY and PRF1 localized predominantly to NK and CD8^+^ T cell compartments, and their expression tracked with activated cytotoxic populations in lesions, underscoring the contribution of these cells to immune surveillance in EM. GNLY and PRF1 did not differ significantly in stromal cells compared with controls, indicating that stromal pathology is not driven by direct modulation of these cytotoxic effectors. Reduced cytotoxic gene programs within immune cells align with immune evasion of lesion cells and continued intraperitoneal expansion [24]. Given their established roles as indices of NK activity, decreased GNLY and PRF1 in lesions provide molecular evidence of impaired NK killing [25, 26]. This impairment coincides with elevated immunosuppressive mediators (for example, TGF‐*β* and IL‐10) and increased expression of inhibitory NK receptors, consistent with NK exhaustion and functional decline [27].

The cell‐resolved alterations in GNLY and PRF1 align with prior observations of diminished NK cytotoxicity in peripheral blood and peritoneal fluid of patients with EM [10, 12, 28]. Receptor‐level changes further support this picture: decreased NKG2D and increased NKG2A have been documented in EM [20]. NKG2D recognizes stress‐induced ligands such as MICA/B and ULBPs [29, 30], reduced receptor expression, and elevated soluble ligands compromise target recognition and killing [30, 31]. Inhibitory signaling via NKG2A–HLA‐E and LILRB1–HLA‐G is enhanced in lesions, dampening NK effector function [32, 33]. Together, these imbalances establish a disabled NK state within the lesion microenvironment [34]. Reports of increased checkpoint molecules such as PD‐1 on NK cells provide additional context for cytotoxic dysfunction in EM [35, 36].

### 4.2. ENTPD1 as a Dual‐Function Mediator

ENTPD1 (CD39) is an ectonucleotidase that hydrolyzes extracellular ATP/ADP to AMP, a precursor of adenosine, thereby contributing to immunosuppressive signaling [37, 38]. In tumors, the CD39/CD73–adenosine axis attenuates antitumor immunity through adenosine receptor engagement and downstream suppression of cytotoxic responses [39, 40]. Reports on the aberrant expression of ectonucleotidases (CD73) in EM indicate involvement of the purinergic metabolic pathway, providing context for ENTPD1‐mediated immunosuppression [34]. In the present study, the elevated expression of ENTPD1 in stromal cells, coupled with its functional promotion of stromal cell invasion and migration, indicates that it both drives lesion behavior and shapes the immune environment. Adenosine signaling attenuates NK degranulation and cytokine production via adenosine receptors, reducing cytotoxic output [41, 42]. These data support a dual‐function model in which ENTPD1 modulates stromal aggressiveness and enforces local immune suppression within a dynamic, interactive microenvironment [43].

Cytokine‐mediated suppression, especially involving TGF‐*β* and IL‐10, constitutes a well‐described dimension of EM immunopathology [44, 45]. Elevated IL‐6 and IL‐10 in peritoneal fluid suppress NK cytotoxicity and bias NK phenotypes toward immune regulation [46, 47]. Against this backdrop, the adenosinergic pathway provides a complementary route to immunosuppression; the ENTPD1 axis and cytokine signals can operate in concert to maintain an immune‐permissive niche that favors ectopic cell survival and proliferation [39, 40, 43]. Therapeutically, the CD39/CD73–adenosine pathway is under active investigation in oncology, offering a conceptual template for mechanism‐guided intervention in EM [48].

At the diagnostic interface, the three‐gene signature (GNLY, PRF1, and ENTPD1) demonstrated robust performance across independent cohorts. Contemporary care still often depends on laparoscopy and is associated with protracted diagnostic timelines, underscoring the need for molecular tools to expedite case identification [49–51]. On the therapeutic front, ENTPD1 represents a compelling target to restore cytotoxic surveillance and restrain stromal invasion. Drug‐repurposing analyses prioritized candidates such as resveratrol, ibuprofen, and indomethacin for pathway‐level modulation of the ENTPD1/GNLY/PRF1 axis, warranting systematic evaluation [52, 53]. More broadly, strategies that restore NK function and rebalance the lesion microenvironment align with mechanism‐informed management aimed at durable symptom control and fertility preservation [54]. Collectively, these findings strengthen the framing of EM as an immune‐mediated disease with actionable molecular hallmarks.

## 5. Limitations

Despite the robustness of our integrative approach, several limitations warrant discussion. First, the in‐house scRNA‐seq dataset was small, which may limit detection of rare subpopulations and introduce patient‐specific bias. Although rigorous batch correction was performed and key findings were validated in larger bulk RNA‐seq cohorts, future studies with expanded single‐cell cohorts are warranted. Second, although bioinformatic analyses strongly implicate ENTPD1‐derived adenosine signaling in NK–cell suppression, we lack direct functional evidence of stromal–immune crosstalk. Finally, ENTPD1’s function was validated only in vitro; in vivo confirmation in animal models and clinical trials will be essential. These limitations notwithstanding, our study provides robust biomarkers and mechanistic insights into EM–associated immune dysregulation.

## 6. Conclusion

In summary, our study integrates multimodal transcriptomic data and functional validation to highlight ENTPD1, along with GNLY and PRF1, as key players in EM pathogenesis. ENTPD1, in particular, emerges as a novel biomarker and functional effector mediating stromal cell migration and immune interactions. These findings offer new insights into the immunopathology of EM and present promising avenues for diagnostic and therapeutic innovation.

## Conflicts of Interest

The authors declare no conflicts of interest.

## Author Contributions

**Wangshu Li**: data curation, funding acquisition, investigation, methodology, writing – original draft. **Fang Wang**: data curation, investigation, methodology, writing – original draft. **Aziz ur Rehman Aziz** and **Xiaohui Yu**: methodology, writing – review and editing. **Kexin** **Zhu**, **Bowen Xu**, and **Juan Nie**: formal analysis, methodology. **Daqing Wang**: conceptualization, data curation, project administration, supervision, writing – original draft. **Chunfang Ha**: funding acquisition, resources, supervision, writing – review and editing.

## Funding

This study was funded by the National Natural Science Foundation of China, Number 82460304; Ningxia Hui Autonomous Region Key Research and Development Program (Project, Number 2023BEG01001); the Dalian City High‐Level Innovative Talent Team Project, Number 2023RG011; and the Dalian City Outstanding Young Science and Technology Talent Program, Number 2023RY020.

## Supporting Information

Additional supporting information can be found online in the Supporting Information section.

## Supporting information


**Supporting Information** Table S1 provides detailed information on the three GEO datasets used in this study, including GEO accession numbers, microarray platforms, sample sizes (endometriosis vs. control), tissue types, and their specific roles as the training cohort, internal validation cohort, or external test cohort.

## Data Availability

The data that support the findings of this study are openly available in GEO at https://www.ncbi.nlm.nih.gov/geo/.
