# Supplementary material for: Single‐Cell Transcriptomics Reveals Biomarkers for NK Cell Dysfunction in Endometriosis‐Associated Immune Dysregulation
Source: Mediators Inflamm. 2026 Feb 3;2026:9028037. doi: 10.1155/mi/9028037 (PMC12866336; doi:10.1155/mi/9028037)
Supplement: Supplementary file 1 — Supporting Information Table S1 provides detailed information on the three GEO datasets used in this study, including GEO accession numbers, microarray platforms, sample sizes (endometriosis vs. control), tissue types, and their specific roles as the training cohort, internal validation cohort, or external test cohort. [file MI-2026-9028037-s001.docx]

| **GEO Accession** | **Platform** | **Samples (Endometriosis vs Control)** | **Tissue Type** | **Role in Study** |
| --- | --- | --- | --- | --- |
| GSE105765 | GPL17027 | 17 (9 EM vs 8 controls) | Eutopic/ectopic endometrium | Training cohort |
| GSE7305 | GPL570 | 20 (10 EM vs 10 controls) | Endometrium | Internal validation |
| GSE6364 | GPL2507 | 37 (20 EM vs 17 controls) | Endometrium | External test cohort |

Supplementary Table S1. Information for each dataset
